# Unlocking patient insights: a prospective study on patient reported outcome measures in thoracic surgery

**DOI:** 10.1186/s13019-026-03950-z

**Published:** 2026-03-13

**Authors:** Marco N. Andreas, Carlo Jurth, Jan C. Schmid, Lisa-Marie Weber, Falk von Dincklage, Aina Lask, Julia Strauchmann, Johann Pratschke, Jens C. Rückert, Jens Neudecker, Tomasz Dziodzio

**Affiliations:** 1 Department of Surgery, Charité–Universitätsmedizin Berlin, Augustenburger Platz 1, 13353 Berlin, Germany; 2 Department of Anesthesiology and Intensive Care Medicine, Charité–Universitätsmedizin Berlin, Charitéplatz 1, 10117 Berlin, Germany; 3 Department of Anesthesia, Intensive Care, Emergency and Pain Medicine, Universitätsmedizin Greifswald, Greifswald, Germany; 4 Berlin Institute of Health at Charité–Universitätsmedizin Berlin, Charitéplatz 1, 10117 Berlin, Germany

**Keywords:** PROMs, Thoracic surgery, Anxiety, Pain, Depression, Post Thoracotomy Pain Syndrome

## Abstract

**Background:**

Patient-reported outcome measures (PROMs) offer the potential for enhancing treatment quality and supporting patient-centered care. However, it remains uncertain how effectively these measures correlate with clinical outcomes.

**Methods:**

This prospective observational cohort study included 107 adult patients undergoing elective thoracic surgery. PROMs assessing anxiety, depression, and pain perception were collected preoperatively and at 6–12 months postoperatively using the Hospital Anxiety and Depression Scale (HADS), the State–Trait Anxiety Inventory (STAI), the Pain Sensitivity Questionnaire (PSQ), and the Pain Catastrophizing Scale (PCS). Postoperative pain was assessed using the Numeric Rating Scale (NRS), and persistent pain at follow-up was defined as post-thoracotomy pain syndrome (PTPS). The primary endpoint was the longitudinal change in PROMs between preoperative assessment and follow-up. Secondary endpoints included subgroup analyses according to the type of surgical access (open and minimally-invasive), the type of locoregional anesthetic technique (epidural anesthesia, intercostal nerve block, local anesthetic infiltration), and the presence of acute and chronic postoperative pain (PTPS).

**Results:**

Patients showed elevated scores at postoperative follow-up, with the exception of the HADS overall score, HADS-anxiety, and STAI, the latter of which demonstrated a significant decrease. No significant differences were found across subgroups based on procedure type, anesthesia method, or PTPS status, with the exception of a significant difference in HADS-depression scores in patients without PTPS.

**Conclusions:**

A decline in physical and psychological well-being was observed 6 to 12 months post-surgery, reflected in increased levels of pain, depression, and anxiety. Our study did not find evidence of an association between the PROMs assessed and variations in surgical procedures, anesthesia methods, or the presence of acute and chronic pain in this cohort.

**Trial registration:**

DRKS: DRKS00017798.

**Supplementary Information:**

The online version contains supplementary material available at 10.1186/s13019-026-03950-z.

## Background

Patient-reported outcome measures (PROMs) are gaining increasing attention within the field of thoracic surgery as crucial tools that provide direct insight into patients’ health status [1]. These measures constitute reports directly from patients may offer invaluable guidance to clinicians, allowing them to better tailor treatment plans beyond classical physician reported parameters. However, ensuring their efficacy necessitates rigorous psychometric testing for both validity and reliability [2].

Thoracic surgeries, whether open or minimally invasive, often result in significant alterations to patients’ physical and psychological well-being. Patients commonly experience a variety of postoperative symptoms, such as pain, fatigue, dyspnea, and anxiety, which can hinder recovery [3]. One of the most challenging complications is post-thoracotomy pain syndrome (PTPS), defined as chronic pain that persists for more than two months after surgery [4]. The prevalence of PTPS is particularly high, with incidences reported as high as 91% after open thoracotomy and up to 33% following minimally invasive procedures [5]. The risk factors contributing to PTPS include surgical approach, perioperative anesthetic management, and the patient’s individual pain sensitivity [6–8].

Psychological factors, such as anxiety and depression, also play a critical role in patients’ postoperative outcomes. A recent meta-analysis involving 14,652 patients revealed that 48% of surgical patients experience preoperative anxiety, with studies showing that anxiety can adversely affect postoperative recovery, leading to complications such as impaired wound healing, increased postoperative pain, and extended hospital stays [9]. Other studies identified anxiety as a potential predictor of survival in patients following thoracic surgery [10]. We hypothesized that PROMs, as a multidimensional tool, can help predicting postoperative complications, particularly PTPS, given its complex pathophysiology and the role of psychological factors. Despite some studies have explored the general use of PROMs in thoracic surgery patients, there has been limited research exploring the specific relationship between PTPS and PROMs. To address this gap, we conducted a prospective study using PROMs on depression, anxiety and pain to investigate the factors contributing to PTPS. The aim was to find out how psychological distress affects acute and chronic pain after thoracic surgery.

## Patients and methods

### Study design

A monocentric, non-blinded prospective observational trial was performed to examine adult patients undergoing elective thoracic surgery at the Department of Surgery - Campus Charité Mitte and Campus Virchow-Klinikum, Charité - Universitätsmedizin Berlin from September 2019 to February 2022. This trial received approval from the institutional ethics board (EA4/088/19) and is registered in the German Clinical Trials Register (DRKS: DRKS00017798) as part of a larger prospective parent study [11]. Written informed consent was obtained from all participants before their inclusion in the study, in accordance with the Declaration of Helsinki guidelines. The aim of this analysis was to investigate the influence of different surgical and anesthetic techniques on changes in pre- and postoperative PROMs and the occurrence of postoperative chronic pain after thoracic surgery. Primary endpoint was the longitudinal change in PROMs from preoperative assessment to follow-up. Secondary endpoints included subgroup analyses by surgical access (open vs. minimally invasive), locoregional anesthetic technique (epidural anesthesia, intercostal nerve block, or local infiltration), and the presence of acute or chronic postoperative pain (PTPS). A STROBE checklist item description with according page numbers can be found in the appendix (Supplementary Figure S1).

### Inclusion criteria

Patients meeting the eligibility criteria were identified during preoperative surgical consultations. Inclusion criteria encompassed all individuals undergoing elective thoracic surgery under general anesthesia. Exclusion criteria comprised individuals under 18 years old, those with a history of prior thoracic surgeries, individuals facing language or communication barriers, or those unable to provide consent.

### Surgical procedure

Operations were carried out through either uniportal Video-Assisted Thoracoscopic Surgery (uVATS), Robotic-assisted Thoracoscopic Surgery (RATS, in 5 port technique), or anterolateral thoracotomy under general anesthesia. The choice of surgical approach was contingent upon factors such as the diagnosis, extent, and location of the resection.

### Analgesia

The utilization of regional anesthesia was in adherence to the hospital’s established standards and independent of the study. Depending on patient and procedural characteristics, either an epidural anesthesia (EDA) was instituted before the procedure, an intercostal block (ICB), or the application of local anesthesia (only in RATS) was performed during the procedure. Peri- and postoperative administration, dosages and types of pain medication were recorded.

### PROMs and pain assessment

Preoperatively and six to twelve months after surgery pain-related psychometric characteristics were queried. Anxiety and depression levels were measured using the State-Trait Anxiety Inventory Questionnaire (STAI), the Pain Sensitivity Questionnaire (PSQ) and the Hospital Anxiety and Depression Scale questionnaire (HADS) [12–14]. The STAI is a psychological assessment tool made up of 40 self-report questions rated on a 4-point scale (range 40 to 160). It evaluates two forms of anxiety: state anxiety (STAI-S, 20 questions) and trait anxiety (STAI-T, 20 questions). Higher scores indicate increased levels of anxiety. The PSQ consists of 17 questions, each rated from 0 (no pain) to 10 (maximum pain). The minimum total score is 0, and the maximum total score is 170, reflecting overall pain sensitivity. The HADS questionnaire is used to assess anxiety and depression in patients with physical illnesses or (possibly psychogenic) physical complaints. It is utilized as a screening tool as well as for dimensional severity assessment, including evaluation over time and measures the extent of anxiety and depressive symptoms through self-assessment over the past week, which is captured on two subscales, each with seven items. The total score ranges from a minimum of 0 to a maximum of 42, with each subscale (anxiety and depression) ranging from a minimum of 0 to a maximum of 21. The total score is used as a measure of general psychological impairment. Pain perception was assessed using the Pain Catastrophizing Scale questionnaire [15]. The PCS is a self-report questionnaire designed to assess catastrophizing in both clinical and non-clinical populations. Catastrophizing is often defined as an excessive negative focus on painful stimuli and significantly influences the experience and management of pain. The PCS includes 13 statements that reflect various thoughts and feelings individuals might have when experiencing pain (each item rated on a 5-point scale, range 0–52). Pre- and postoperative pain evaluation utilized the Numeric Rating Scale (NRS) [16], conducted during the preoperative screening, immediately upon the patients’ return to consciousness in the recovery room or intensive care unit, daily during the morning ward rounds until discharge and 6 to 12 months after the operation. At follow-up, patients were additionally asked to specify the localization of pain. PTPS was defined as the presence of persistent pain with an NRS score > 1 at 6–12 months after surgery, provided that the pain was clearly localized to the surgical site, thereby excluding pain unrelated to the thoracic procedure.

### Statistical analysis

Descriptive variables are expressed as median (minimum-maximum range) or counts and percentages. Questionnaire scores are reported as mean ± standard deviation. Normal distribution for two compared variables was confirmed using the D’Agostino-Pearson test. If both variables were normally distributed and independent, a t-test for independent samples with Welch’s correction was applied; otherwise, the Mann-Whitney U test was used. For variables with a dependency, a t-test for paired samples (normally distributed) or the Wilcoxon test (non-normally distributed) was conducted. A chi-squared test was performed to assess the comparative values among distinct populations. Statistical significance was set at *p* < 0.05 for two-sided tests. Missing data at individual time points were not imputed. If data were available for only one of the two time points (preoperative or postoperative), the available data were included in the corresponding analysis. Paired comparisons were performed only for cases with complete data at both time points. Unpaired comparisons were conducted at each time point based on the available data. Statistical analysis utilized IBM SPSS Statistics (Version 29.0.0.0, IBM Coop., Armonk, USA), while GraphPad Prism 9.1.2 (GraphPad Software Inc., La Jolla, CA, USA) was employed for both statistical analysis and visualization.

## Results

### Study population

**Fig. 1:**
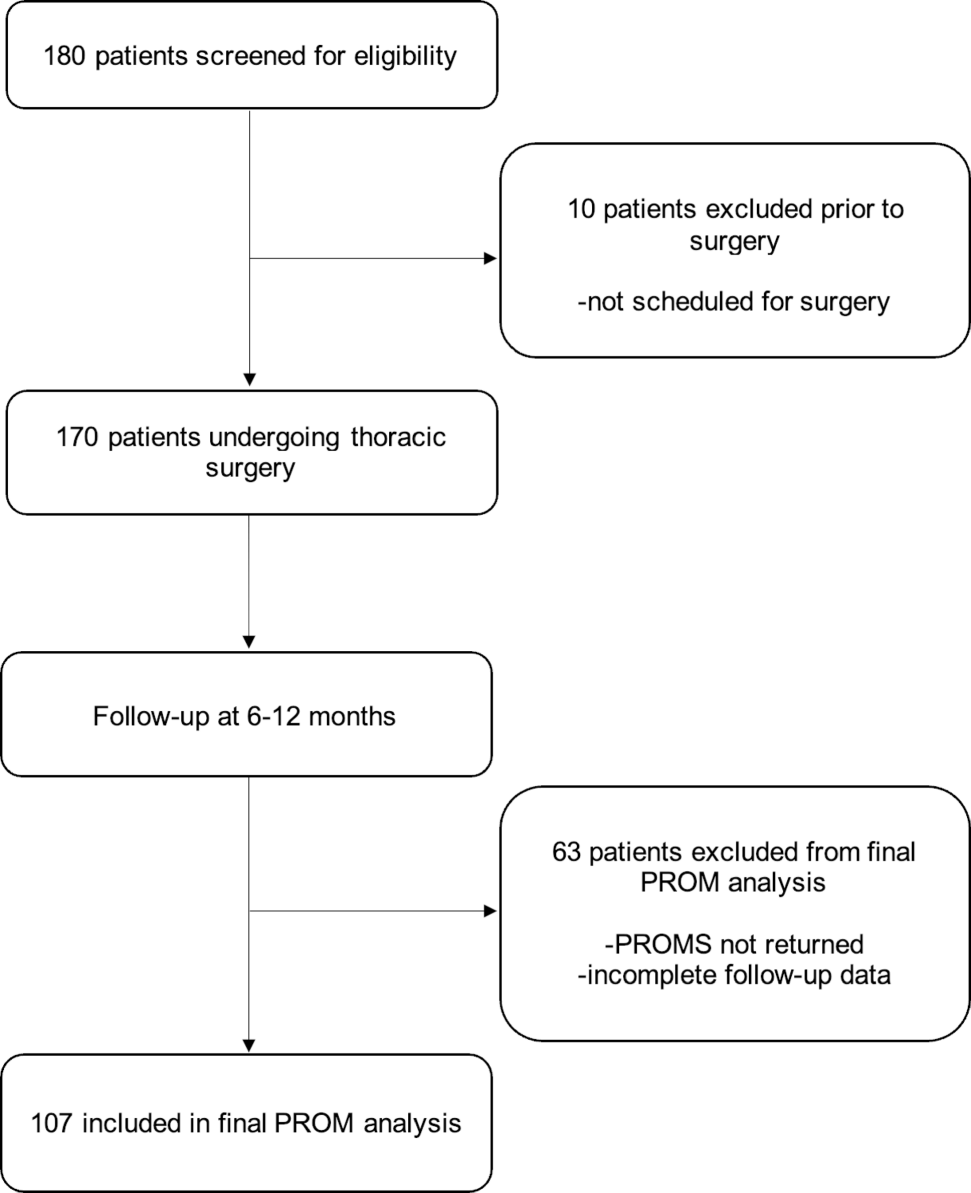
STROBE (Strengthening the Reporting of Observational Studies in Epidemiology) diagram of the study population

Out of the initially 180 screened patients, 170 (94.4%) were successfully scheduled for surgery. Of this group, 107 (59.4%) underwent both pre- and postoperative PROM assessments. The study population is illustrated by a STROBE (Strengthening the Reporting of Observational Studies in Epidemiology) diagram in Fig. 1. Among the 107 patients who completed the assessments, 78 (72.9%) were operated via a minimally invasive (MI) approach, either VATS or RATS, while 29 (27.1%) underwent surgery via thoracotomy (open surgery – OS). Regarding additional locoregional anesthetic treatment, 32 patients (29.9%) received epidural anesthesia (EDA), 58 (54.2%) received an intercostal nerve block (ICB), and 15 patients (14.0%; those undergoing RATS) received local anesthetic infiltration at the port sites. In two cases (1.9%, both RATS-procedures), no additional locoregional anesthetic technique was applied beyond general anesthesia. A comprehensive set of descriptive statistics for the patients is available in Table 1 for further reference.

**Table 1:** Descriptive statistics pertaining to the patient cohort are presented as median (minimum-maximum range) for continuous variables and as n (%) for categorical variables

Parameter	
Gender (m/f)	59 (55.1%) / 48 (44.9%)
Age (years)	63 (20–82)
Pack years (years)	40 (3–120)
FEV1 (l)	2.2 (0.5–4.5)
FEV1 (%)	81 (19–128)
ASA classification	3 (1–4)
ICU (hours)	0 (20–82)
Procedure Thoracotomy VATS RATS	29 (27.1%) 61 (57.0%) 17 (15.9%)
Type of locoregional anesthesia technique None* Epidural Intercostal Local	2 (1.9%) 32 (29.9%) 58 (54.2%) 15 (14.0%)
Resection type Lobectomy Segmentectomy Wedge Resection Other^†^	21 (19.6%) 41 (38.3%) 30 (28.0%) 15 (14.0%)

* “None” refers to procedures performed under general anesthesia without additional locoregional analgesic techniques (e.g., epidural anesthesia, intercostal nerve block, or local anesthetic infiltration prior to skin incision). This applied to two robotic-assisted procedures

^†^ “Other” includes pleurectomy, mediastinal lymph node dissection, and resection of mediastinal tumors

m = male, f = female, FEV1 = forced one-second-capacity, ASA = American Society of Anesthesiologists, ICU = intensive care unit, VATS = Video-assisted Thoracoscopy, RATS = Robotic-assisted Thoracoscopy

### PROMs comparison before and after surgery

**Fig. 2:**
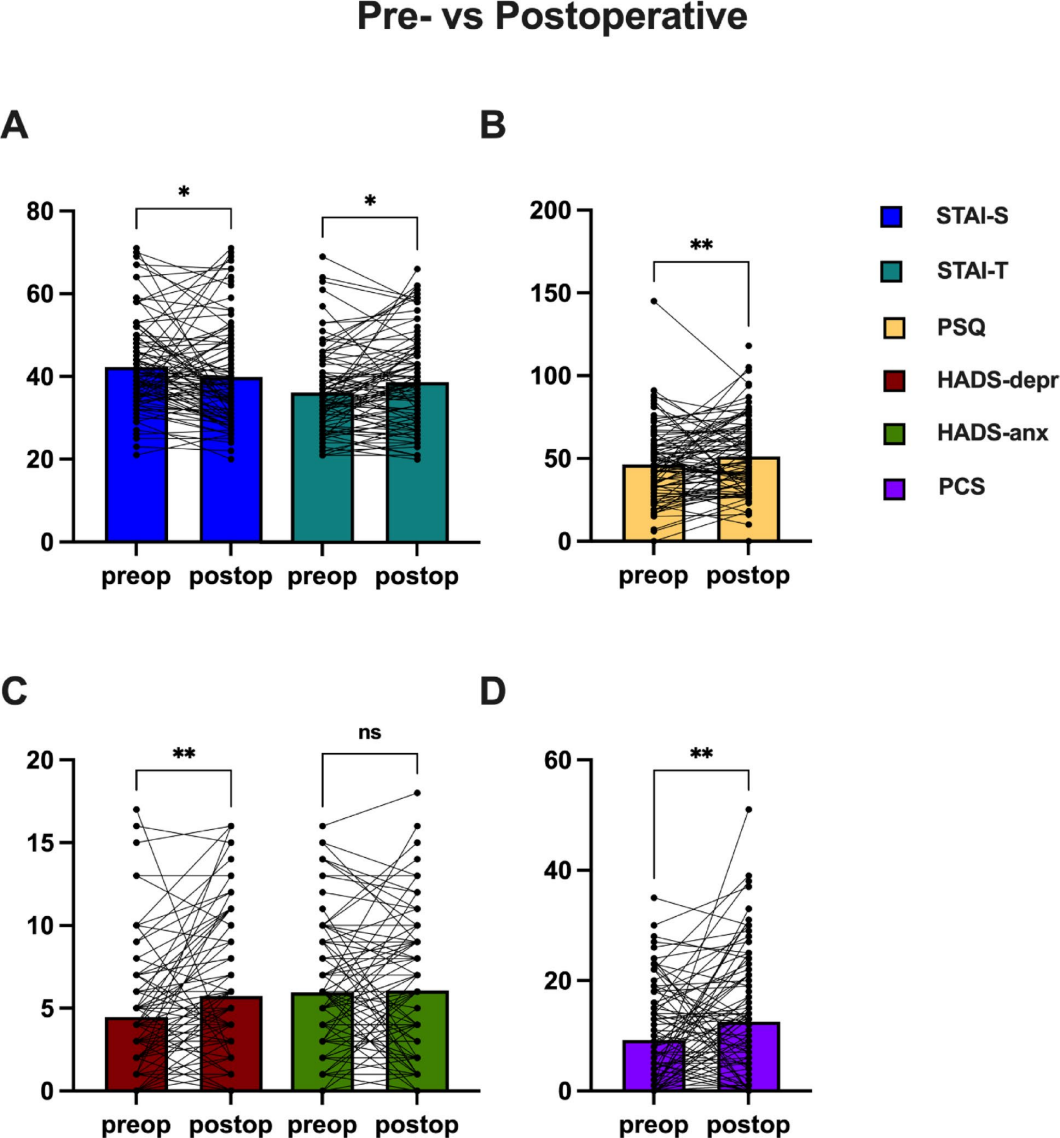
The Patient-Reported Outcome Measure (PROM) test results were juxtaposed, examining data collected both prior to and 6–12 months post-operation. Bar graphs illustrate the means ± standard deviation. Individual patient values are shown as dots, with paired pre- and postoperative measurements connected by lines (* = *p* < 0.05, ** = *p* < 0.01)

The pre- and postoperative test results for all patients were analyzed (Fig. 2). A notable decrease in the STAI-S test score was observed postoperatively (42.32 ± 10.86 pre-op, 39.92 ± 12.21 post-op, *p* = 0.0144), contrasting with a higher postoperative score in STAI-T (36.17 ± 10.09 pre-op, 38.70 ± 11.06 post-op, *p* = 0.019). Similarly, both PSQ and PCS scores exhibited significant increases in the postoperative phase (PSQ: 46.41 ± 22.42 pre-op, 51.3 ± 22.37 post-op, *p* = 0.0060; PCS: 9.21 ± 8.93 pre-op, 12.57 ± 11.36 post-op, *p* = 0.0065). While the overall HADS test results showed no statistically significant differences (10.43 ± 6.88 pre-op, 11.81 ± 7.91 post-op, *p* = 0.0568, Supplementary Figure S2), the depression component demonstrated a noteworthy elevation postoperatively (4.47 ± 3.62 pre-op, 5.74 ± 4.33 post-op, *p* = 0.0014). Conversely, HADS-anxiety displayed no significant differences (5.96 ± 3.94 pre-op, 6.08 ± 4.25 post-op, *p* = 0.9618).

### PROMs subgroup analysis

**Fig. 3:**
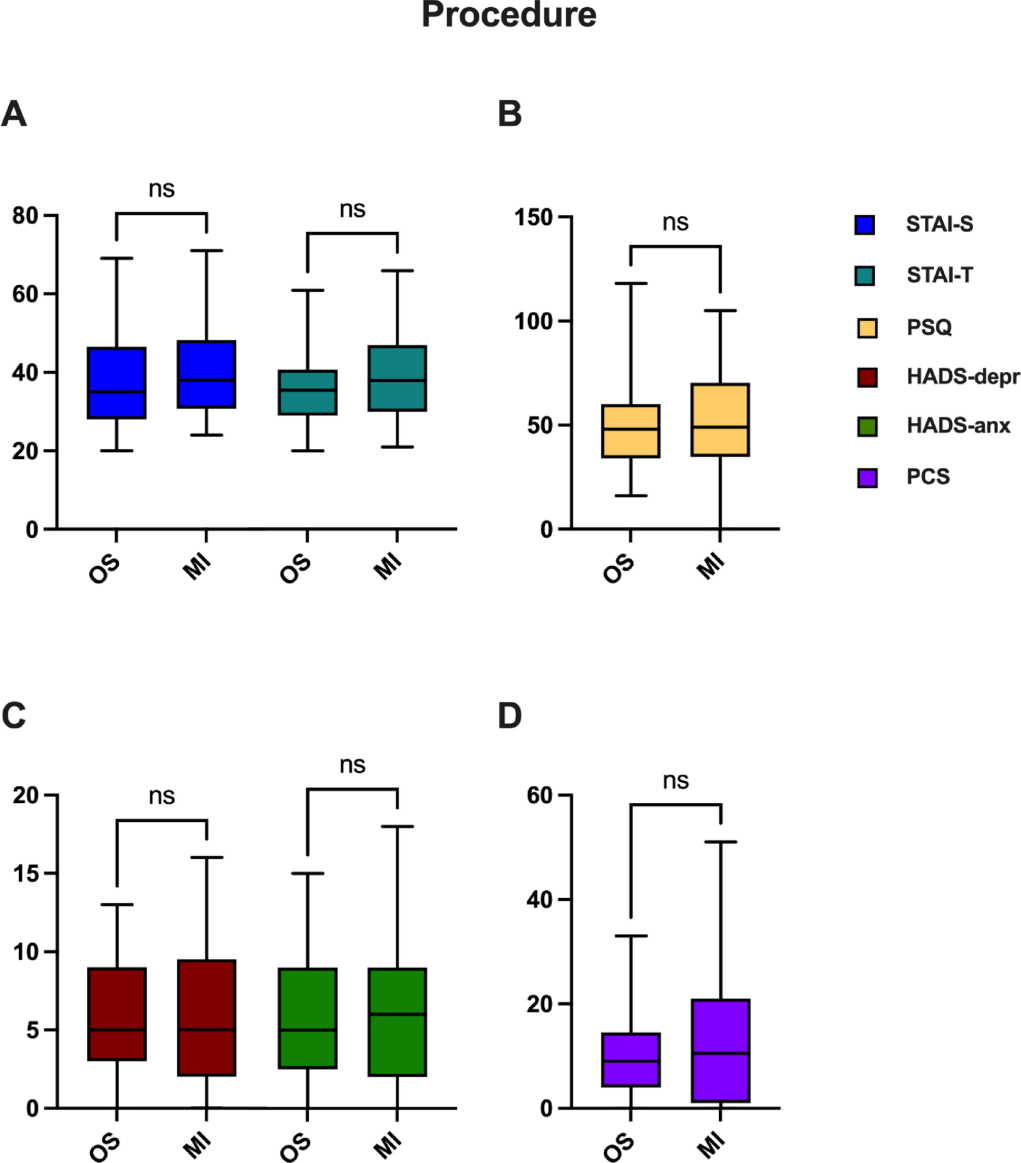
Patients’ PROMs test results 6–12 months after the operation compared with regard to surgical approach: open surgery (OS) or minimally invasive surgery (MI). Bars represent mean ± standard deviation

**Fig. 4:**
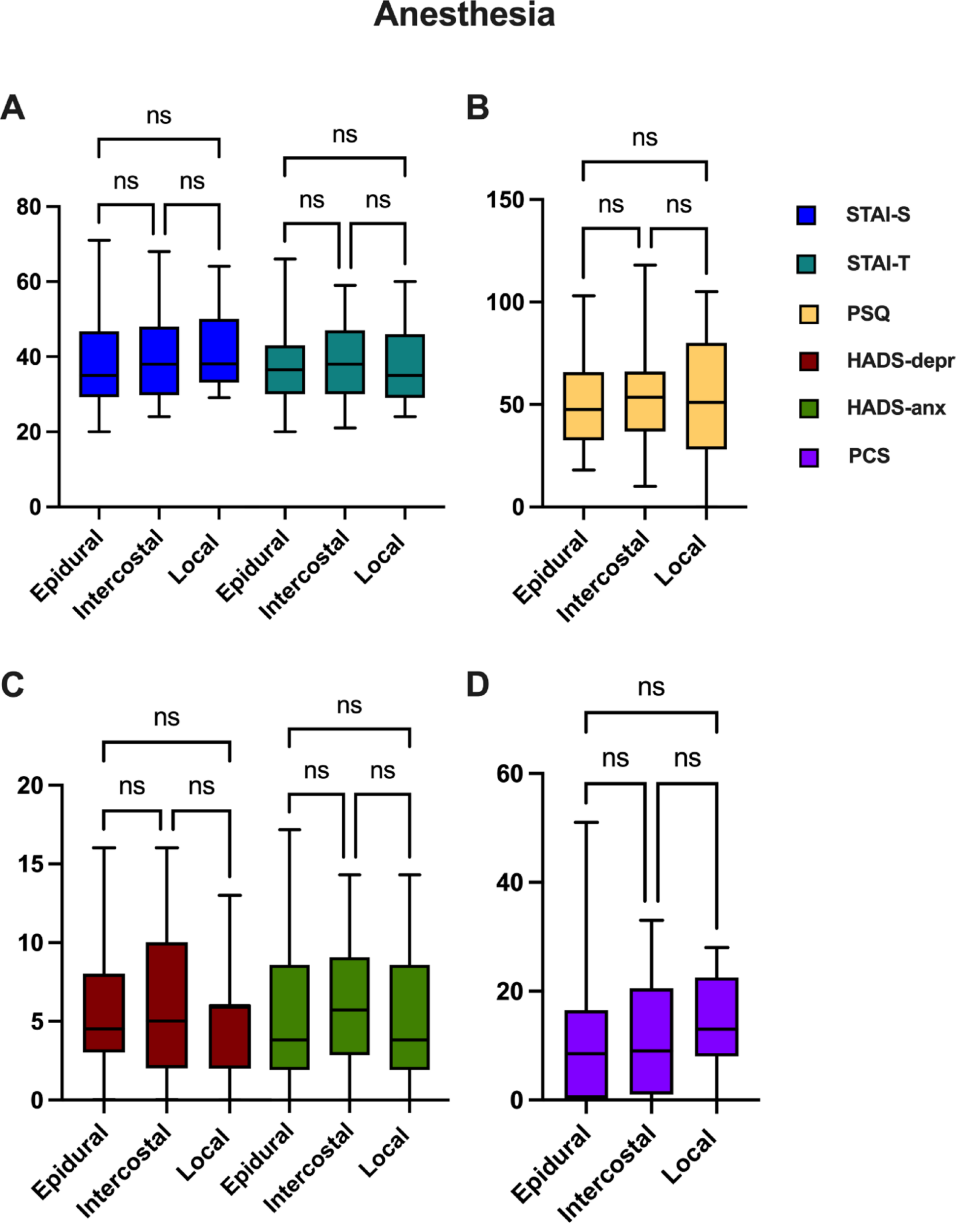
Patients’ PROMs test results 6–12 months after the operation were compared with regard to the anesthetic technique (epidural block, intercostal nerve block, local anesthesia) during the operation. Bars represent mean ± standard deviation

**Fig. 5:**
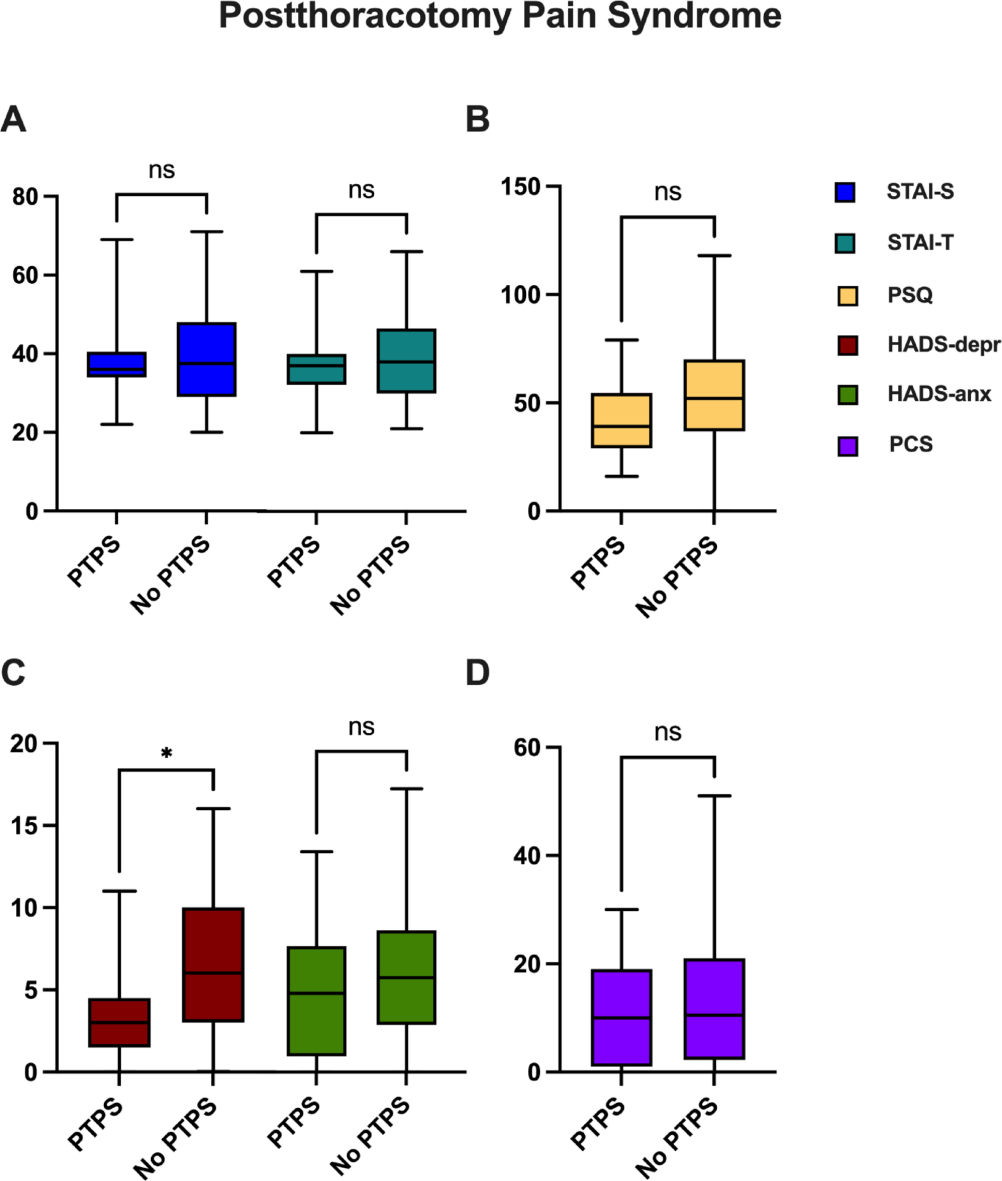
Patients’ PROMs test results 6–12 months after the operation were compared in relation to the presence or absence of Post Thoracotomy Pain Syndrome (PTPS). Bars represent mean ± standard deviation

To delve into potential disparities in test outcomes, a subgroup analysis was conducted focusing on follow-up results (6–12 months post-surgery), examining procedure type (Fig. 3), anesthesia method (Fig. 4), and the presence or absence of PTPS (Fig. 5). Evaluation of procedure subgroups did not provide evidence of statistically significant differences (STAI-S: *p* = 0.223; STAI-T: *p* = 0.1327; PSQ: *p* = 0.5194; PCS: *p* = 0.8139; HADS: *p* = 0.9449; HADS-depression: *p* = 0.8694; HADS-anxiety: *p* = 0.9535), albeit a subtle inclination for the MI subgroup to exhibit slightly elevated scores, particularly in anxiety-related assessments such as STAI-S, STAI-T and HADS anxiety.

Similarly, we did not observe evidence of statistically significant differences among the three anesthesia approaches (STAI-S: *p* = 0.6693; STAI-T: *p* = 0.9328; PSQ: *p* = 0.8139; PCS: *p* = 0.5392; HADS: *p* = 0.6160; HADS-depression: *p* = 0.8506; HADS-anxiety: *p* = 0.5566), although the ICB subgroup demonstrated a tendency for higher scores across all tests, except for the PCS, where the local subgroup exhibited the highest mean score with 60.21.

Regarding the subgroup analysis of patients with and without PTPS, those without PTPS exhibited significantly higher scores in the depression component of the HADS (*p* = 0.016). Other comparisons yielded non-significant results, with a slight trend toward higher test scores in the PTPS-free group (STAI-S: *p* = 0.9142; STAI-T: *p* = 0.4882; PSQ: *p* = 0.0720; PCS: *p* = 0.6354; HADS: *p* = 0.0527; HADS-anxiety: *p* = 0.2415).

## Discussion

We conducted a prospective cohort study involving 107 patients who underwent thoracic surgery. To assess pain sensitivity, pain management, anxiety, and depression, we utilized four validated scores: STAI, PSQ, PCS, and HADS. In the follow-up period, patients showed significantly higher scores in STAI, PSQ, and PCS, while the HADS overall score and HADS-anxiety did not exhibit notable changes. A subgroup analysis, which examined procedure type, anesthesia method, and the presence or absence of PTPS, did not provide evidence of statistically significant differences, except for higher HADS-depression scores in patients without PTPS. These findings suggest complex interrelationships between psychological distress and pain management.

### Comparison between preoperative and follow-up assessments

We observed increased PSQ and PCS scores during follow-up, indicating heightened pain sensitivity and insufficient self-management of pain. Current literature supports the association between higher pain sensitivity scores and increased pain levels, but due to the lack of comparable studies on postoperative pain sensitivity and management, a definitive conclusion cannot be made [17].

Preoperative anxiety, which is a common occurrence among surgical patients, can significantly influence postoperative outcomes [17]. The prevalence of preoperative anxiety in thoracic surgery patients can range widely, depending on factors such as the type of surgery, the patient’s sex, and age. For example, Ju et al. reported a preoperative anxiety rate of 48.4% in lung cancer patients undergoing video-assisted thoracoscopic surgery (VATS) [18]. In our study, trait anxiety (measured by STAI-T) significantly increased postoperatively, while state anxiety (measured by STAI-S) showed a slight decline. Trait anxiety refers to an individual’s predisposition to perceive situations as threatening, characterized by a persistently heightened baseline arousal. In contrast, state anxiety represents temporary emotional responses to specific situations [19, 20]. Both HADS-anxiety and STAI-S predominantly assess state anxiety [21]. The lack of significant changes in HADS-anxiety suggests that state anxiety remains relatively unaffected by thoracic surgery, while trait anxiety may worsen over time. In our institution, psychological distress is routinely screened during inpatient care using the NCCN Distress Thermometer, with established referral pathways for patients exceeding predefined thresholds [22]. This structured perioperative screening may contribute to the stable HADS-anxiety scores and the decrease in STAI-S, indicating effective management of acute, surgery-related anxiety. However, comparable structured psychological screening is not routinely implemented during long-term postoperative follow-up, which may help explain the observed increase in trait anxiety at 6–12 months. Taken together, our findings, which align with prior research, suggest that thoracic surgery has a more substantial impact on long-term anxiety (trait anxiety) than on transient emotional responses (state anxiety) [23]. This aligns with findings from another prospective trial that reported no differences in HADS-anxiety pre- and postoperatively [24].

Notably, postoperative depression, measured using the HADS-depression subscale, also significantly increased during follow-up, consistent with previous findings from studies such as that of Park et al., which demonstrated heightened depression scores after thoracic procedures [24]. These results underscore the importance of integrating psychological assessments into routine clinical follow-up, allowing for early intervention for patients in need of supportive care.

Interestingly, the observed increase in all scores in our study does not appear to be driven solely by individual patients scoring higher postoperatively. Rather, a more nuanced pattern emerges upon closer inspection, with some patients showing declines while others experience increases, resulting in an overall collective rise.

### Subgroup analysis

In the subgroup analysis, we compared patients based on their surgical procedure - open surgery versus minimally invasive techniques (VATS and robotic-assisted thoracic surgery, or RATS). No evidence of statistically significant differences was observed in our study between the groups. This finding contrasts with the existing literature, which suggests that open thoracotomy is more likely to result in higher levels of postoperative anxiety and depression [24].

Similarly, we did not find evidence that anesthesia technique was associated with PROM results at follow-up. Although the anesthesia subgroups (epidural, intercostal block, local anesthesia) showed some divergent tendencies, no consistent pattern or clear preference emerged; notably, the local anesthesia subgroup comprised only 15 patients, limiting statistical power. To further contextualize these findings beyond the technical approach alone, we examined early postoperative pain intensity (NRS) and postoperative analgesic requirements, which are summarized in Supplementary Table S1. While previous prospective studies have shown that higher levels of acute postoperative pain are associated with an increased risk of chronic pain development, early postoperative pain scores and analgesic use in our cohort were largely comparable between patients with and without PTPS [25]. This observation underscores the multifactorial nature of chronic pain development and may help explain the absence of clear associations with long-term PROM outcomes in our study.

For example, Ho et al. demonstrated in our study, PTPS was examined for its impact on physical and psychological well-being. While one might expect pain sensitivity scores to be predictive of PTPS, we did not find a clear evidence for an association between these scores and the development of PTPS, consistent with findings by Hasan et al., who did not identify a link between pain and anxiety in patients one month after thoracic surgery [17]. The similarity between our cohort and that of Hasan et al.‘s study may be due to the heterogeneity of patients undergoing thoracic surgery for various reasons. Most existing literature, which indicates anxiety and depression as risk factors for postoperative pain due to their impact on the physiological stress axis, focuses on more homogeneous patient groups [24, 26, 27]. Significant effects are often observed in short-term evaluations, where higher anxiety scores are linked to higher pain levels immediately after surgery. In our long-term follow-up, the lack of evidence for an association between PROMs and surgical or anesthetic procedures could suggest that these scores are influenced by multiple factors, including non-medical ones (such as financial stress or the loss of a loved one).

Interestingly, patients without PTPS tended to score higher in overall PROM measures, particularly with respect to depressive symptoms, a finding that at first glance appears counterintuitive. One might expect patients with persistent postoperative pain to report higher levels of psychological distress. However, an alternative explanation may be that patients without ongoing pain are more attentive to their psychological well-being or to other life stressors that are not dominated by pain-related concerns, potentially resulting in higher reported depression scores. In contrast, patients experiencing PTPS may primarily focus on somatic symptoms, which could attenuate the reporting of depressive symptoms in PROM-based assessments. This observation highlights the complex interplay between physical symptoms and psychological outcomes and suggests that the absence of pain does not necessarily equate to better mental health. Importantly, this finding should be interpreted with caution due to the limited number of patients who developed PTPS in our cohort (*n* = 17). Nevertheless, it underscores the need for future studies with larger sample sizes to further explore the relationship between postoperative pain, psychological well-being, and patient-reported outcomes.

### Clinical implications

The postoperative increase in PROM scores indicates a clinically relevant psychological and pain-related burden and highlights opportunities for targeted intervention. Routine PROM-based follow-up may enable early identification of at-risk patients, particularly when predefined score thresholds or meaningful score changes trigger automated alerts to caregivers. In the future app-based PROM platforms could facilitate longitudinal monitoring beyond hospital discharge and support timely referral to psycho-oncological or pain management services. Integrating such PROM-driven alert systems into clinical workflows may help translate patient-reported data into actionable postoperative care.

### Limitations

This prospective cohort study includes a substantial number of patients undergoing various thoracic procedures (such as open surgery, VATS, and RATS) and receiving different anesthetic approaches (including epidural, intercostal block, and local anesthesia). We used four different PROMs to evaluate postoperative impairments, focusing on pain, depression, and anxiety, to capture the multidimensional physical and psychological well-being of patients.

However, the study has some limitations. Despite using standardized tests, interpreting PROMs can be complex, with individual scores potentially challenging to compare. A significant limitation lies in reduced comparability to other studies, as many employ quality-of-life (QoL) questionnaires. Our study, by contrast, emphasized physical and psychological well-being measures specifically tailored to assess PTPS, which may restrict comparisons with broader QoL-focused research.

The drop-out rate was relatively high at 33%. In most cases, postoperative PROMs were not completed, which may be attributable to the method of data collection—by mail—compared to the preoperative PROMs, which were completed in person during the hospital stay. The use of app-based questionnaires could help reduce this drop-out rate in future follow-up studies.

Our subgroup analyses face limitations due to small sample sizes (e.g., RATS *n* = 17, local anesthesia *n* = 15, PTPS *n* = 17), impacting statistical power. Additionally, some subgroups were heterogeneous, such as the combined analysis of RATS and VATS, which use distinct port configurations, complicating statistical interpretation.

Additionally, we did not differentiate between patients with benign and malignant diseases, which may have introduced bias, particularly regarding the potential impact of malignancy on patients’ quality of life.

Further, we did not assess PROMs between the operation and the 6–12-month follow-up period, nor did we perform time-stratified analyses within this follow-up window. This limits insights into potential time-associated effects and confounding influences - such as tumor progression or recurrence, intercurrent illness, or psychosocial stressors - as well as into the influence of anesthetic or surgical procedures on PROM outcomes during the intermediate postoperative period.

Lastly, perioperative variables such as operative duration, length of hospital stay, blood loss, reoperation, and postoperative medical complications were not included in the comparative analyses. Although these factors may influence patient-reported well-being, major postoperative events were rare in our cohort, and further stratification would likely have resulted in statistically unstable subgroups. Future studies designed to capture perioperative course and recovery-related parameters may provide additional insights.

## Conclusions

PROMs provide valuable insight into patient-centered outcomes following thoracic surgery. In this prospective study, we observed evidence of statistically significant longitudinal changes, including a decrease in state anxiety and increases in trait anxiety and depressive symptoms at 6 to 12 months postoperatively. At the same time, we did not find evidence of statistically significant differences in PROMs across surgical procedures, anesthetic approaches, or PTPS status. The lack of evidence for associations suggests that broader PROMs may be influenced by multiple medical and non-medical factors, underscoring the need for more specialized questionnaires tailored to the unique psychological and physical stressors associated with thoracic surgery.

## Supplementary Information

Supplementary Material 1

Supplementary Material 2

Supplementary Material 3

## Data Availability

The data underlying this article will be shared on reasonable request to the corresponding author.

## Abbreviations

HADS: Hospital Anxiety and Depression Scale
ICB: Intercostal nerve block
MI: Minimally invasive
NCCN: National Comprehensive Cancer Network
NRS: Numeric rating scale
OS: Open surgery
PCS: Pain Catastrophizing Scale
EDA: epidural anesthesia
PROMs: Patient Reported Outcome Measures
PSQ: Pain Sensitivity Questionnaire
PTPS: Post Thoracotomy Pain Syndrome
QoL: Quality of Life
RATS: Robotic-assisted Thoracoscopy
VATS: Video-assisted Thoracoscopy
STAI: State Trait Anxiety Inventory
STAI-S: State Trait Anxiety Inventory – State anxiety
STAI-T: State Trait Anxiety Inventory – Trait anxiety
