# Supplementary material for: Unlocking patient insights: a prospective study on patient reported outcome measures in thoracic surgery
Source: J Cardiothorac Surg. 2026 Mar 13;21:173. doi: 10.1186/s13019-026-03950-z (PMC13064385; doi:10.1186/s13019-026-03950-z)
Supplement: Supplementary file 1 — Supplementary Material 1 [file 13019_2026_3950_MOESM1_ESM.pdf]

STROBE Statement—checklist of items that should be included in reports of observational studies

	Item No.	Recommendation	Page No.	Relevant text from manuscript
**Title and abstract**	1	(*a*) Indicate the study's design with a commonly used term in the title or the abstract	2	"This prospective observational cohort study included 107 adult patients undergoing elective thoracic surgery."
		(*b*) Provide in the abstract an informative and balanced summary of what was done and what was found	2-3	Abstract (Methods, Results, Conclusions)
**Introduction**				
Background/rationale	2	Explain the scientific background and rationale for the investigation being reported	3-4	Background, paragraphs 1–3 (PROMs relevance, PTPS, psychological factors)
Objectives	3	State specific objectives, including any prespecified hypotheses	4	"The aim was to find out how psychological distress affects acute and chronic pain after thoracic surgery."
**Methods**				
Study design	4	Present key elements of study design early in the paper	4-5	Methods: Study design
Setting	5	Describe the setting, locations, and relevant dates, including periods of recruitment, exposure, follow-up, and data collection	4	Methods: Study design "…Charité – Universitätsmedizin Berlin from September 2019 to February 2022."
Participants	6	(*a*) Give the eligibility criteria, and the sources and methods of selection of participants. Describe methods of follow-up	5	Methods: Inclusion criteria
		(*b*) For matched studies, give matching criteria and number of exposed and unexposed	n/a	n/a
Variables	7	Clearly define all outcomes, exposures, predictors, potential confounders, and effect modifiers. Give diagnostic criteria, if applicable	5-6	Methods: PROMs and pain assessment

Data sources/ measurement	8*	For each variable of interest, give sources of data and details of methods of assessment (measurement). Describe comparability of assessment methods if there is more than one group	5-6	Methods: PROMs and pain assessment (STAI, PSQ, PCS, HADS, NRS)
Bias	9	Describe any efforts to address potential sources of bias	13-15	Discussion: Limitations
Study size	10	Explain how the study size was arrived at	4-5 & 7-8	Methods: Study design & Results: Study population
Quantitative variables	11	Explain how quantitative variables were handled in the analyses. If applicable, describe which groupings were chosen and why	7	Methods: Statistical analysis "Descriptive variables are expressed as median (minimum–maximum range) […] Questionnaire scores as mean ± SD."
Statistical methods	12	(*a*) Describe all statistical methods, including those used to control for confounding	7	Methods: Statistical analysis
		(*b*) Describe any methods used to examine subgroups and interactions	7	Methods: Statistical analysis (subgroup analyses by surgery, anesthesia, PTPS)
		(*c*) Explain how missing data were addressed	7	Methods: Statistical analysis "Missing data at individual time points were not imputed…"
		(*d*) If applicable, explain how loss to follow-up was addressed	4-5 & 7-8	Methods: Study design & Results: Study population
		(*e*) Describe any sensitivity analyses	n/a	n/a
**Results**				
Participants	13*	(a) Report numbers of individuals at each stage of study—eg numbers potentially eligible, examined for eligibility, confirmed eligible, included in the study, completing follow-up, and analysed	7	"Out of the initially 180 screened patients, 170 (94.4%) were successfully scheduled for surgery. Of this group, 107 (59.4%) underwent both pre- and postoperative PROM assessments.

		(b) Give reasons for non-participation at each stage	7	as mentioned under (a) &Figure 1
		(c) Consider use of a flow diagram	7	Figure 1
Descriptive data	14*	(a) Give characteristics of study participants (eg demographic, clinical, social) and information on exposures and potential confounders	7-8	Results: Study population, Table 1
		(b) Indicate number of participants with missing data for each variable of interest	8	Study population, Figures 2-5
		(c) Summarise follow-up time (eg, average and total amount)	7	Figure 1
Outcome data	15*	Report numbers of outcome events or summary measures over time	8	Results: PROMs comparison before and after surgery
Main results	16	(*a*) Give unadjusted estimates and, if applicable, confounder-adjusted estimates and their precision (eg, 95% confidence interval). Make clear which confounders were adjusted for and why they were included	8-9	Results: PROMs comparison before and after surgery, PROMs subgroup analysis, Figures 2-5
		(*b*) Report category boundaries when continuous variables were categorized	6-7	PTPS defined as NRS > 1 at 6–12 months, localized to the surgical site
		(*c*) If relevant, consider translating estimates of relative risk into absolute risk for a meaningful time period	n/a	n/a
Other analyses	17	Report other analyses done—eg analyses of subgroups and interactions, and sensitivity analyses	8-9	Results: PROMs subgroup analysis, Figures 2-5
**Discussion**				
Key results	18	Summarise key results with reference to study objectives	9	Discussion, first paragraph
Limitations	19	Discuss limitations of the study, taking into account sources of potential bias or imprecision. Discuss both direction and magnitude of any potential bias	13-15	Discussion: Limitations
Interpretation	20	Give a cautious overall interpretation of results considering objectives, limitations, multiplicity of analyses, results from similar studies, and other relevant evidence	9-15	Discussion: entire section except Limitations; Conclusion
Generalisability	21	Discuss the generalisability (external validity) of the study results	13-15	Discussion: Limitations & Conclusion
**Other information**				
Funding	22	Give the source of funding and the role of the funders for the present study and, if applicable, for the original study on which the present article is based	17	Funding statement

*Give information separately for exposed and unexposed groups.

Note: An Explanation and Elaboration article discusses each checklist item and gives methodological background and published examples of transparent reporting. The STROBE checklist is best used in conjunction with this article (freely available on the Web sites of PLoS Medicine at http://www.plosmedicine.org/, Annals of Internal Medicine at http://www.annals.org/, and Epidemiology at http://www.epidem.com/). Information on the STROBE Initiative is available at www.strobe-statement.org.
