# Supplementary material for: Unlocking patient insights: a prospective study on patient reported outcome measures in thoracic surgery
Source: J Cardiothorac Surg. 2026 Mar 13;21:173. doi: 10.1186/s13019-026-03950-z (PMC13064385; doi:10.1186/s13019-026-03950-z)
Supplement: Supplementary file 2 — Supplementary Material 2 [file 13019_2026_3950_MOESM2_ESM.docx]

**Supplementary material**

**Tab. S1:** Potential risk factors for Post Thoracotomy Pain Syndrome displayed as descriptive analysis

|  |  | Overall | No PTPS | PTPS |
| --- | --- | --- | --- | --- |
| N |  | 107 | 90 | 17 |
| Chronic pain (%) | no | 62 (64.6) | 50 (62.5) | 12 (75.0) |
|  | yes | 34 (35.4) | 30 (37.5) | 4 (25.0) |
| Minimally-invasive approach (%) | no | 29 (27.1) | 24 (26.7) | 5 (29.4) |
|  | yes | 78 (72.9) | 66 (73.3) | 12 (70.6) |
| Regional anesthesia (%) | none | 2 (1.9) | 1 1.1) | 1 (5.9) |
|  | EDA | 32 (29.9) | 26 (28.9) | 6 (35.3) |
|  | ICB | 58 (54.2) | 48 (53.3) | 10 (58.8) |
|  | Local | 15 (14.0) | 15 (16.7) | 0 (0.0) |
| Complications (%) | none | 72 (67.3) | 59 (65.6) | 13 (76.5) |
|  | local | 17 (15.9) | 16 (17.8) | 1 (5.9) |
|  | general | 13 (12.1) | 11 (12.2) | 2 (11.8) |
|  | both | 5 (4.7) | 4 (4.4) | 1 (5.9) |
| Additional pain medication (%) | no | 61 (57.0) | 51 (56.7) | 10 (58.8) |
|  | yes | 46 (43.0) | 39 (43.3) | 7 (41.2) |
| NRS before recovery room (median [IQR]) |  | 1.00  [0.00, 3.00] | 1.00  [0.00, 3.00] | 0.00  [0.00, 2.00] |
| NRS after recovery room (median [IQR]) |  | 2.00  [0.00, 4.00] | 2.00  [0.00, 4.00] | 1.00  [0.00, 3.50] |
| NRS > 2 recovery room (%) | no | 55 (61.1) | 46 (61.3) | 9 (60.0) |
|  | yes | 35 (38.9) | 29 (38.7) | 6 (40.0) |
| NRS day 1 (median [IQR]) |  | 2.00  [0.00, 3.00] | 2.00  [1.00, 4.00] | 1.00  [0.00, 2.00] |
| NRS day 1 while coughing (median [IQR]) |  | 3.00  [1.00, 4.00] | 3.00  [1.00, 4.00] | 3.00  [1.00, 3.00] |

Abbreviations: IQR = interquartile range, NRS = numeric rating scale, PTPS = Post Thoracotomy Pain Syndrome, EDA = epidural catheter, ICB = intercostal block.
