# Supplementary figures and images for: Unlocking patient insights: a prospective study on patient reported outcome measures in thoracic surgery
Source: J Cardiothorac Surg. 2026 Mar 13;21:173. doi: 10.1186/s13019-026-03950-z (PMC13064385; doi:10.1186/s13019-026-03950-z)

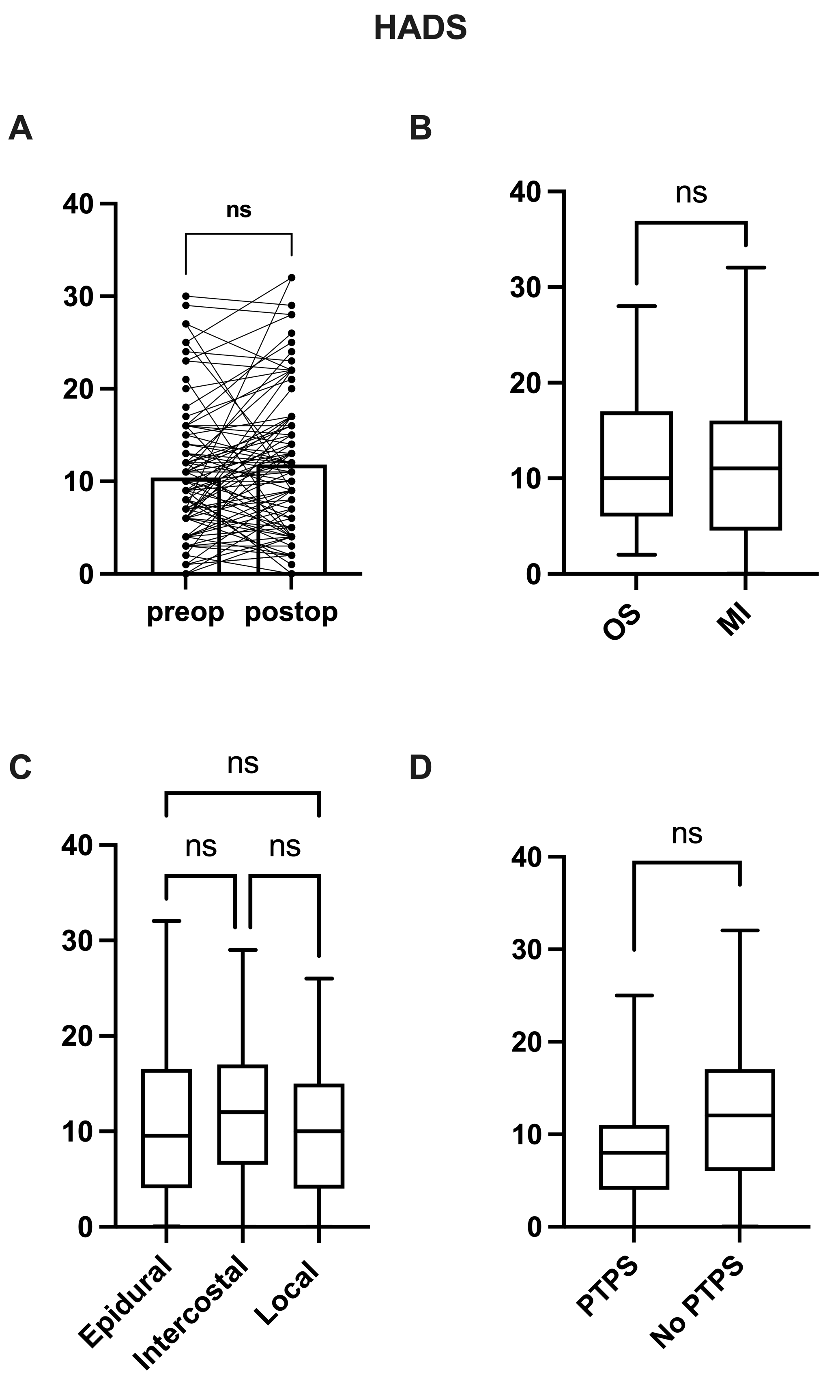

Supplement: Supplementary file 3 — Supplementary Material 3 [file 13019_2026_3950_MOESM3_ESM.tiff]
